# Cephalometric Analysis, Severity Malocclusion, and Orthodontic Treatment Need Using IOTN in Deaf Children

**DOI:** 10.1055/s-0041-1735936

**Published:** 2021-11-24

**Authors:** Noengki Prameswari, Herniyati Herniyati, Bambang Sucahyo, Arya Brahmanta, Meralda Rossy Syahdinda

**Affiliations:** 1 Department of Orthodontic, Faculty of Dental Medicine, Universitas Hang Tuah, Surabaya, Indonesia; 2 Department of Orthodontic, Faculty of Dental Medicine, Universitas Jember, Jember, Indonesia

**Keywords:** deaf children, cephalometric analysis, index of orthodontic treatment need, malocclusion

## Abstract

**Objectives** Studies associated with deaf children's malocclusion and their treatment need are still very rare. Therefore, cephalometric analysis with the ability to access the skeletal, dental, and soft tissues can be used to score the severity of malocclusion and index of orthodontic treatment need (IOTN) in deaf children. This study examined the use of cephalometric analysis, severity malocclusion, and orthodontic treatment need in deaf students at special need school type B (SLB-B) *Tunarungu Karya Mulia* in Surabaya using IOTN along with investigating IOTN correlation with the result of dental cephalometric analysis and dental health component (DHC) and aesthetic component (AC) in IOTN index.

**Material and Methods** Sample data consisted of 33 students between the ages of 8 to 12 years old and never had any orthodontic treatment. This investigation applied the indices from IOTN, in which DHC had 10 malocclusions, and AC with the aesthetic anterior dentition comprising 10 color photographs and different dental attractiveness levels. In addition, scores were chosen from the worst feature, with the data analyzed at a significant correlation test of 0.05%.

**Result** There was no skeletal abnormality in deaf children. It displayed the highest number of malocclusion severity scores, while the DHC assessment showed the moderate and severe categories. Based on AC evaluation, the highest numbers of malocclusion severity were found in good and moderate category in terms of teeth arrangement and aesthetic.

**Conclusion** There was a correlation between the dental cephalometric analysis in deaf children and treatment need using IOTN with AC and DHC.

## Introduction

Orthodontics refers to the supervision, guidance, and correction of both developing and developed dentofacial structures. 1 It has a broad scope that comprises a variety of malocclusions, which involves misalignment of the upper and lower sets of teeth or dental and jaw arches, affecting the masticatory and facial aesthetics. 2 Malocclusions range from simple to complex. Its prevalence varies according to the geographical base, population, ethnicity, age, sex, and urbanization, and it can be overcome by utilizing secure procedures that are also applicable to the type that requires orthognathic surgery. 3 It is usually diagnosed in patients with typical health conditions such as cleft lip and palate, Down's syndrome, as well as other diseases; however, it also emerges due to the direct outcome of a disorder or associated ailment. 4 Furthermore, it is also detected in children with a hearing impairment, leading to the communication problems that affect certain aspects of their growth, development, and ability. 5 Speech disorder is a type of condition or communicative behavior characterized by difficulty in the production of speech sounds. This occurs due to certain abnormalities in the shape and structure of the speech organs, which consist of politicizes, malocclusions, anomalies, deviations, or congenital defects such as the thick tongue or minimal growth of frenulum. 6

Cephalometric analyses have been used as the standard measuring the craniofacial growth and development. Besides orthodontic and skeletal growth analysis, lateral cephalograms are also often applied to measure various cranial parameters. It also offers valuable information concerning the growth and development of intracranial parameters, irrespective of the fact that it is a standardized radiography technique. 7 Furthermore, cephalometric radiography is an essential tool in the diagnosis and treatment of malocclusions underlying the skeletal discrepancies. Thus, the utilization of serial cephalometric radiographs makes it possible to examine and predict the progress of orthodontic treatment as well as the surgical outcome of dentofacial deformity. 8

Correspondingly, the concepts of orthodontic treatment are based on three aspects, which are objective signs, subjective symptoms, and social views. Objective signs include abnormalities of teeth that deviate from normal, subjective symptoms are self-perceived anomalies that require treatment, and social views include the general opinion that a person's malocclusion needs to be treated. The last one depends on the local and social culture. 9 Moreover, children with hearing impairment need attention and tools that aid the treatment of these abnormalities to overcome their communication problems. Direct communication is difficult because the children usually cannot understand spoken communication. 10 Research works on jaw growth and the functions of the teeth and oral mucosa in deaf children are rare, even though their desire to get treatment is quite high. 11

Simultaneously, orthodontics has applied several special indices in order to determine the severity of the malocclusion. 12 An example is the index of orthodontic treatment need (IOTN) which classifies malocclusions based on the abnormalities of individual dental structures and the acceptance of aesthetic imperfections to prioritize orthodontic treatment. IOTN measures the needs of children under the age of 18 years for epidemiological surveys or the success of treatment. It has the following advantages: it is easy to use, simple, of short duration, clinically justified according to the needs, clearly differentiates several levels, as well as statistically accountable. 13 In addition, this index displays an acceptable degree of conformity between examiners; therefore, it is implemented for epidemiological surveys. Correspondingly, it is appropriate for this study, because it was discovered that children with hearing loss, communication problems, and adaptability, usually have family protection factors and sometimes require different interpersonal treatments. On the other hand, there are several other types such as treatment priority index (TPI), occlusal index (OI), index of complexity, outcome, and need (ICON), dental aesthetic index (DAI); however, IOTN is more complete in terms of components than the others. 14 It consists of two components, which are dental health component (DHC) and aesthetic component (AC), which are performed together to determine the patients' needs. This index is also designed to discover which patients have the most severe malocclusions by implementing DHC followed by AC. 13 15 Moreover, studies concerning deaf children and associated with cephalometric analysis and IOTN index are still limited. Thus far, this is the only study to investigate the severity of malocclusion and orthodontic treatment in deaf children as well as the correlation between the dental cephalometric analysis with DHC and AC in IOTN.

## Materials and Methods

### Study Subject and Design

This research protocol obtained the ethical clearance committee of Faculty of Dentistry, Hang Tuah University, with appointment number KEPK/EC/001/NALA.RSGM/X/2019. This study was a case-control study with cross-sectional design conducted from October to November 2019 with the help of the total sampling method. The population in this study were 33 special need school type B (SLB-B) *Tunarungu Karya Mulia* students in Surabaya, East Java, Indonesia, in compliance with the following inclusion sampling criteria: they were between the ages of 8 to 12 years (growth age), males and females (they were not distinguished based on sex), and never received any orthodontic treatment. The exclusion criteria were deaf children with other syndromes such as Down's syndrome, Usher syndrome, and cleft lip/palate. All patients' guardians were asked to fulfill written informed consent in order for their children to be subjects in this study voluntarily.

The carried out research procedures included the following: (1) recording the general identity of the samples by writing the name, age, and gender on the survey card, (2) providing the information on the procedures carried out in this research, such as photography, cephalometric and any impression of a study model for each sample, (3) conducting the cephalometric analysis of images measuring the angles of angle between Sella–N–A point (SNA), angle between Sella–N–B point (SNB) and angle between A point–N–B point (ANB), and the soft tissue, (4) interpreting the results, and, (5) observing and measuring the severity of malocclusion in the study model and the photo samples using DHC and AC in IOTN components, respectively.

**Fig. 1:**
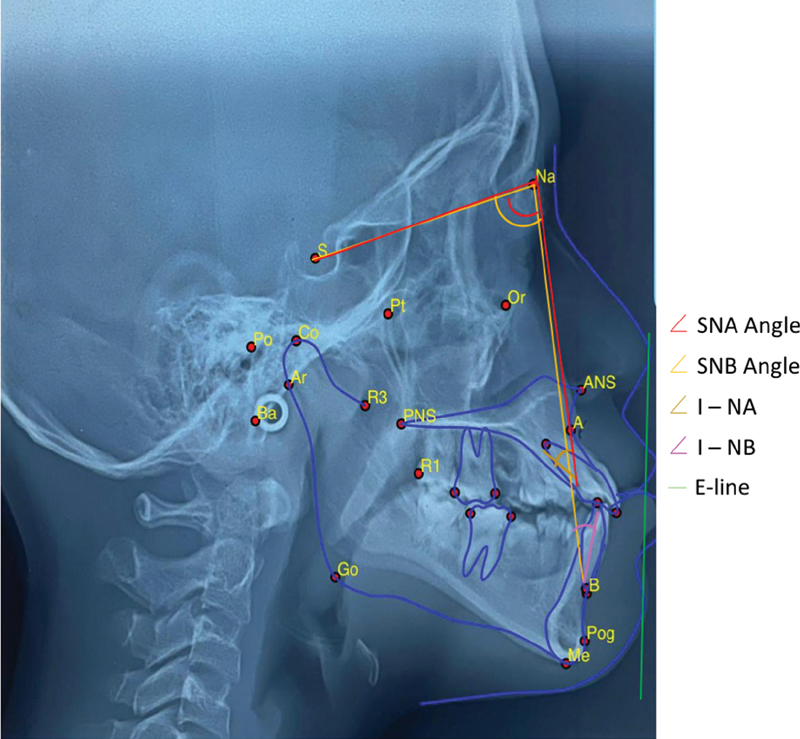
The anatomical landmarks of cephalometric analysis that were investigated is as follows: SNA Angle determined by points Sella (the center of sella turcica), Nasion (the most anterior point of the frontonasal suture) and Point A (the innermost point on the contour of the maxilla between the anterior nasal spine and the alveolar crest); SNB angle determined by point Sella, Nasion and Point B (the most posterior point in the concavity along the anterior border of the symphysis); I-NA (angle formed by the intersection of the upper incisor axis and the NA line); I-NB (angle formed by the intersection of the lower incisor axis and the NB line); E-line joining the soft tissue pogonion and pronasale and its relation with upper and lower lips. 16

All samples were positioned in the cephalostat with the sagittal plane at right angles to the X-rays, the Frankfort plane was parallel to the floor, and the centric occlusion of the teeth and the lips were lightly sealed together. The digital tracings (Webceph, Korea) as well as all the measurements were performed by a third investigator, an experienced orthodontist with many years of cephalometric experience. Then, the selected landmarks were traced with bilateral structures, and their average was calculated to generate a single structure or landmark. Next, all measurements were carried out manually and entered into an Excel spreadsheet for statistical evaluation.

Skeletal and dental cephalometric measurements utilized in this research calculated the angles of SNA anteroposterior position of the maxilla relative to the anterior cranial base, SNB anteroposterior position of the mandible relative to the anterior cranial base, ANB, which is the difference between SNA and SNB angles and defines the mutual relationship in the sagittal plane of the maxillary and mandibular bases, the upper incisor to NA (1-NA), which is the angle formed between the long axis of upper central incisor and the anteroposterior position and, finally, lower incisor to NB (1-NB), which is the angle formed between long axis of lower central incisor and anteroposterior position of the mandible. The analysis of the interincisal and soft tissues applied E Line ( Fig. 1 ). 16

Moreover, a transparent plastic ruler was used to measure DHC, while a photo color was used to measure the aesthetic component. Any abnormalities were diagnosed based on severe deviations, while 9 DHCs were recorded by examining the following occlusal traits: missing teeth, overjet, crossbite, displacement, overbite (MOCDO). All the five grades of DHC were defined by implementing criteria; in addition, grading was conducted, based on DHC concerning the development of IOTN. 13 14

The aforementioned five grades for DHC were no need for orthodontic treatment, minor need for orthodontic treatment, moderate/borderline need for orthodontic treatment, orthodontic treatment required, and great orthodontic treatment required. The grade for DHC of IOTN was recommended for individuals with severe malocclusion traits. 13 14 Furthermore, AC measured the aesthetic impairment and justified the treatment on social-psychological grounds. Therefore, it ranked malocclusion in terms of various occlusal traits detected in a person's dental health as well as a perceived aesthetic impairment to identify those that were likely to be benefitted from an orthodontic treatment. 13 14 In addition, AC scale was arranged, based on the photographs of teeth captured from the anterior view of 10000 on 12-year-old children with an average of 6 photos being taken for visual comparison. Then, 10 photos were chosen as illustrations, and they were also arranged into 10 scales, showing variations in the dentition of the teeth. 15

The degree of severity and treatment requirements of AC was categorized as follows: a) scale 1 to 4 showed the condition of the teeth arrangement with good aesthetics or mild abnormalities; therefore, it required minor or no treatment, b) scale 5 to 7 showed the state of aesthetic tooth structure which required borderline or moderate treatment, and c) scale 8 to 10 showed the poor aesthetic condition of the teeth; therefore, it required treatment. 1 13

In this research, data was examined and scored according to the indices of IOTN, which consisted of two components, DHC and AC, respectively. The most severe scores were analyzed descriptively, and their percentages were calculated using frequency distribution. Furthermore, the interrelation between DHC, orthodontist's aesthetic opinion, and dental cephalometric analysis was determined using the Spearman correlation with significant value of 0.05. The statistical analysis of Shapiro–Wilk test and Levene's test was carried out by means of statistical package for social science (SPSS) version 20.0 (IBM corporation, Illinois, Chicago, US).

## Results

The cephalometric examination in deaf children was conducted using the Steiner analysis. The cephalometric analysis conducted on deaf children showed no abnormality in their skeleton, however, 82% of those in skeletal class I had I-NA protrusion, 1-NB retrusion, and hypertonus of upper and lower lips ( Table 1 ).

**Table 1:** Descriptive data of cephalometric analysis in deaf children

Parameter	Normal value	Values in deaf children	Number	Percentage
SNA	79°–89°	79°–89°	30	91
		< 79°	3	9
SNB	74°–89°	74°–89°	30	91
		< 74°	3	9
ANB	0°–4°	0°–4° skeletal (class I)	27	82
		< 0° (skeletal class 1)	3	9
		> 4° (skeletal class II)	3	9
1-NA	26°	26° (normal)	9	27
		> 26° (protrusion)	15	46
		< 26° (retrusion)	9	27
1-NB	29°	29° (normal)	3	9
		> 29° (protrusion)	12	36
		< 29° (retrusion)	18	55
Intrinsical	118°	118° (normal)	6	18
		> 118° (upright)	18	55
		< 118° (protrusion)	9	27
Soft-tissue analysis	Upper lips 2 mm behind the E-line	Normal	18	55
		Hypertonus lips	15	45
	Lower lips 2 mm behind the E-line	Normal	12	36
		Hypertonus lips	21	64

Abbreviations: ANB, angle between A point–N–B point; SNA, Sella–N–A point; SNB, Sella–N–B point.

Corresponding to this research, the highest numbers of malocclusion severity score using DHC assessment were in the grades 3 and 4 with the percentage of 36% in moderate and severe malocclusion ( Table 2 ). In relation to AC evaluation, the most severe malocclusion was expressed on the scale of 1 to 7, with 64% of the sample having good and moderate teeth arrangement as well as aesthetic impairment ( Table 3 ). Based on the treatment needs assessed by implementing DHC, it revealed that 91% of the samples, which comprised 36% and 55% of the respondents required moderate (borderline) and absolute treatment, respectively. On the contrary, based on the assessment conducted with AC, 63% of the sample, which consisted of 36% and 28% of the respondents, needed moderate (borderline) and absolute treatment ( Table 4 ).

**Table 2:** Malocclusion severity in deaf children using the DHC component of IOTN index

Malocclusion severity	Malocclusion type	Number	Percentage
Grade 2: mild malocclusion/little need requirement	2.d: Displacement of teeth > 1 mm but ≤ 2 mm	3	9
Grade 3: Moderate malocclusion/borderline need	3.d: Displacement of teeth > 2 mm but ≤4 mm 3.f: Increased and incomplete overbite without gingival or palatal trauma	12	36
Grade 4: Severe malocclusion/treatment need	4d: Severe displacements of teeth > 4 4.g: Less extensive hypodontia requiring prerestorative orthodontics or orthodontic space closure to obviate the need for a prosthesis 4.j: Partially erupted teeth, tipped and impacted against adjacent teeth	12	36
Grade 5: Severe malocclusion/great treatment need	5.i: Impeded eruption of teeth (apart from 3rd molars) due to crowding, displacement, the presence of supernumerary teeth, retained deciduous teeth, and any pathological cause	6	19
Total		33	100

Abbreviations: DHC, dental health component; IOTN, index of orthodontic treatment need.

**Table 3:** Severity malocclusion in deaf children using IOTN AC component

Severity malocclusion	Number	Percentage
Scale 1–4: good teeth arrangement and aesthetic/no need treatment	12	36
Scale 5–7: moderate teeth arrangement and aesthetic/borderline need	12	36
Scale 8–10: poor teeth arrangement and aesthetic/treatment need	9	28
Total	33	100

Abbreviations: AC, aesthetic component; IOTN, index of orthodontic treatment need.

**Table 4:** Treatment needs in deaf children using IOTN

Treatment need		DHC component		AC component	
No need treatment		3 (9%)		12(36%)	
Malocclusion	Borderline treatment	30 (91%)	12 (36%)	21 (63%)	12 (36%)
	Need treatment		18 (55%)		9 (28%)
Total		33 (100%)		100%	

Abbreviations: AC, aesthetic component; DHC, dental health component; IOTN, index of orthodontic treatment need.

The result data of the ordinal variables have a large number of levels; hence, the Spearman test was applied to examine the correlation between the dental cephalometric analysis and the treatment using both the components of AC and DHC in IOTN. This research had a statistic significant of 0.00 with a correlation coefficient of – 0.793 between ANB (skeletal parameter/ sagittal plane of the maxillary and mandibular bases) and DHC, – 0.637 between ANB and AC, and 0.866 between DHC and AC. Therefore, a considerable association between two variables existed. Moreover, a significance level of slightly less than 5% presented that the probability of the relationship was accidentally discovered to be approximately 5 in a 100. Meanwhile, Rs value of – 0.793 and – 0.637 suggested a fairly strong negative relationship, while 0.866 also determined a fairly strong relationship ( Table 5 ).

**Table 5:** Spearman correlation test result between dental cephalometric analysis and DHC AC IOTN index

	Skeletal (ANB)	Dental (interincisal)	DHC	AC
Skeletal correlation coeff.	1.0	0.028	–0.793	–0.637
Sig		0.878	0.00	0.00
Dental correlation coeff.	0.028	1.0	0.63	0.88
Sig	0.878		0.729	0.627
DHC correlation coeff.	– 0.793	0.63	1.0	0.866 a
Sig.	0.00	0.729		0.000
AC correlation coeff.	– 0.637	0.088	0.866 a	1.0
Sig.	0.00	0.627	0.000	

Abbreviations: AC, aesthetic component; ANB, angle between A point–N–B point; DHC, dental health component; IOTN, index of orthodontic treatment need.

a information: significant at *p* > 0.05.

## Discussion

A cephalometric analysis conducted on deaf children showed that there was no abnormality in their skeletons, however, 82% of the respondents in skeletal class I had I-NA protrusion, 1-NB retrusion, as well as hypertonus in the upper and lower lips. This means that the skeleton is not affected by deaf impairment. Abnormalities detected in the dental analysis were caused by protrusion of the upper incisors and retrusion of the lower incisors. 17 Furthermore, children with tongue adaptation show greater lower lips activity. 18 The tip of the tongue slightly overlays the lower incisors when it is at rest and protrudes beyond it during the “s” production in defective speakers. 19 Therefore, the adaptation capacity of the oropharyngeal structure contributes fundamentally. This adaptation is based on the children's characters, level of intelligence, muscle control, emotional state, and social condition. 20

In this present study, we found that the highest numbers of malocclusion severity score using DHC assessment were in the grades 3 and 4 in moderate and severe malocclusion. In relation to AC evaluation, the most severe malocclusion was expressed on the scale of 1 to 7, having good and moderate teeth arrangement as well as aesthetic impairment. According to treatment needs assessed by implementing DHC, it was revealed that most samples required moderate (borderline) and absolute treatment. On the contrary, based on the assessment conducted with AC, most samples needed moderate (borderline) and absolute treatment. This showed that deaf children need orthodontic treatment, particularly in the cases of malocclusion, compared to the aesthetic of their anterior teeth. In this study, the classification of malocclusion was not specifically examined, even though class I, II, or III malocclusions can show sagittal and vertical changes that occur in the growth period, which correlate with the dental condition. 21 Consistently, a research conducted by Ajami also argued that most children with disabilities had class I malocclusion based on the classification of angle. 22

Moreover, children with hearing impairment usually complain of the ear pain and fever, and they also have allergies and often experience the respiratory infections such as sinusitis and tonsillitis. 23 Various airway diseases also cause the breathing through the mouth, which affects their dentocraniofacial growth, resulting in the inappropriate eruption of teeth in the oral cavity. 24 This was consistent with the studies carried out on four children where contact point displacement (CPD) > 4 mm. The lack of dentocraniofacial growth and development also can prevent the teeth from eruption. 12 25 This was in accordance with the results from the research conducted on hypodontia, which was discovered to be less extensive and required restoration treatment or orthodontic space closure, thereby eliminating the need to conduct prostheses in four children. 26 In addition, any dental craniofacial growth affects tooth eruption; according to Klein, this relates to the results from the research conducted on the partial eruption, tipping, and impaction of the next tooth in one child, because the late eruption is associated with crammed, excess, and retention of deciduous teeth as well as the other pathological abnormalities in six children. 27

Additionally, children with hearing loss mostly have poor oral hygiene compared to the normal ones. 23 The caries examination conducted using DMF-T displayed that deaf students classified within the age group of 11 to 12 years old were 4.17 higher than normal ones (3.46). A similar circumstance was detected in deaf students within a group age of 14 to 16 years who expressed caries at 5.53 higher than nonhearing impaired ones (3.80); thus, they needed oral health-promoting intervention to assist them to learn the favorable oral health practices and skills in tooth brushing along with the ability to choose a proper diet. 12 Meanwhile, difficult eating, unsatisfactory diet, and interrupting meals were significantly correlated with malocclusion severity. 26 This is consistent with the results from an investigation conducted on numerous children with hearing impairment, with nine children possessing cases of crowding (CPD). Moreover, one of the causes in crammed teeth is caries, which is also consistent with the research conducted by Ajami which stated that an examination conducted on children with disabilities using the oral health index-simplified (OHI-S) index showed that they possessed a high caries rate (81.7%); therefore, they are not adequately taken care of with poor OH status (49.4%). 22

Related to measurement results on the severity of malocclusion by applying AC, the highest percentage was recorded and categorized in the scale of 1 to 4 for teeth arrangement, with aesthetically good in 12 children (36%) who needed minor or no treatment, while another 36% were included in the scale of 5 to 7 for teeth arrangement with moderate aesthetics, which required the moderate or borderline care, and the remaining 28% was categorized within a scale of 8 to 10 for both poor teeth arrangement and aesthetics, which needed an orthodontic treatment. The result of AC was much likely to decide the necessity for orthodontic treatment and provide plans for health prevention lists by setting up the priorities. 27

Finally, the difficulty encountered in this research was the impression of a study model conducted among 33 children. This was because children with hearing loss generally found it difficult to focus when they were asked to speak. Therefore, sensitive series of explanations were carried out among children who were sensitive and prone to vomit. This was consistent with the research conducted by Alsmark that deaf children need special communication such as intonation and clear articulation, with the addition of the need to communicate with their parents when necessary. 11 Finally, the difficulties encountered in determining the severity of malocclusion, based on AC, involved the requirement of two or more people to achieve an objective result.

## Conclusion

Based on the results from the research conducted at SLB-B *Tunarungu Karya Mulia* students in Surabaya, using cephalometric analysis, no abnormality was detected in the skeletal system of deaf children. However, 82% of the sample were categorized in skeletal class I with I-NA protrusion, 1-NB retrusion, and hypertonus in the upper and lower lips. The highest numbers of malocclusion severity score was in accordance with DHC assessment, which were in the grades 3 and 4 (moderate and severe malocclusion) with the percentage of 36%. Meanwhile, AC evaluation was used to detect the most severe malocclusion, which was categorized on a 1 to 7 scale of good and moderate in teeth arrangement and aesthetic with the percentage of 64%. Additionally, there was a correlation between dental cephalometric analysis and treatment need using AC and DHC of IOTN. This study suggest that deaf children need preventive or interceptive orthodontic treatment for correcting facial aesthetic and good occlusion. However, further study is still needed to investigate complexity outcome and needs of orthodontic treatment using ICON.
